# The Affinity of Hemoglobin for Oxygen Is Not Altered During COVID-19

**DOI:** 10.3389/fphys.2021.578708

**Published:** 2021-04-12

**Authors:** Thomas Gille, Lucile Sesé, Eric Aubourg, Emmanuelle E. Fabre, Florence Cymbalista, Kayaththiry Caroline Ratnam, Dominique Valeyre, Hilario Nunes, Jean-Paul Richalet, Carole Planès

**Affiliations:** ^1^Service de Physiologie et Explorations Fonctionnelles, Hôpital Avicenne, GHUPSSD, Assistance Publique—Hôpitaux de Paris, Bobigny, France; ^2^Inserm UMR 1272 “Hypoxie et Poumon,” UFR SMBH Léonard de Vinci, Université Sorbonne Paris Nord, Bobigny, France; ^3^CNRS, CEA, Astroparticule et Cosmologie, Université de Paris, Paris, France; ^4^Laboratoire de Biochimie, Hôpital Avicenne, GHUPSSD, Assistance Publique—Hôpitaux de Paris, Bobigny, France; ^5^Inserm UMR 978 ASIH, UFR SMBH Léonard de Vinci, Université Sorbonne Paris Nord, Bobigny, France; ^6^Laboratoire d’Hématologie-Biologie, Hôpital Avicenne, GHUPSSD, Assistance Publique—Hôpitaux de Paris, Bobigny, France; ^7^Service de Pneumologie, Centre de Référence Maladies Pulmonaires Rares, Hôpital Avicenne, GHUPSSD, Assistance Publique—Hôpitaux de Paris, Bobigny, France

**Keywords:** COVID-19, SARS-CoV-2, hemoglobin-oxygen affinity, P50, gas exchange, gas transport, hemolysis, anemia

## Abstract

**Background:** A computational proteomic analysis suggested that SARS-CoV-2 might bind to hemoglobin (Hb). The authors hypothesized that this phenomenon could result in a decreased oxygen (O_2_) binding and lead to hemolytic anemia as well. The aim of this work was to investigate whether the affinity of Hb for O_2_ was altered during COVID-19.

**Methods:** In this retrospective, observational, single-center study, the blood gas analyses of 100 COVID-19 patients were compared to those of 100 non-COVID-19 patients. Fifty-five patients with carboxyhemoglobin (HbCO) ≥8% and 30 with sickle cell disease (SCD) were also included (“positive controls” with abnormal Hb affinity). P_50_ was corrected for body temperature, pH, and PCO_2_.

**Results:** Patients did not differ statistically for age or sex ratio in COVID-19 and non-COVID-19 groups. Median P_50_ at baseline was 26 mmHg [25.2–26.8] vs. 25.9 mmHg [24–27.3], respectively (*p* = 0.42). As expected, P_50_ was 22.5 mmHg [21.6–23.8] in the high HbCO group and 29.3 mmHg [27–31.5] in the SCD group (*p* < 0.0001). Whatever the disease severity, samples from COVID-19 to non-COVID-19 groups were distributed on the standard O_2_-Hb dissociation curve. When considering the time-course of P_50_ between days 1 and 18 in both groups, no significant difference was observed. Median Hb concentration at baseline was 14 g.dl^–1^ [12.6–15.2] in the COVID-19 group vs. 13.2 g.dl^–1^ [11.4–14.7] in the non-COVID-19 group (*p* = 0.006). Among the 24 COVID-19 patients displaying anemia, none of them exhibited obvious biological hemolysis.

**Conclusion:** There was no biological argument to support the hypothesis that SARS-CoV-2 could alter O_2_ binding to Hb.

## Introduction

In December 2019, a novel coronavirus named severe acute respiratory syndrome coronavirus 2 (SARS-CoV-2) emerged in the Chinese city of Wuhan. The related coronavirus disease (COVID-19) rapidly spread worldwide during the following months, straining healthcare resources in many countries (Yu et al., 2020; Zhu et al., 2020). This pandemic urged the scientific community to quickly uncover and deliver information about the disease. Therefore, a substantial number of preprint articles have been made available, sparking a debate on whether they constitute reliable sources of scientific data (Smyth et al., 2020). Among them, an *in silico* modeling of molecular docking suggested that some structural and non-structural viral proteins might bind to hemoglobin (Hb) in several spots (Wenzhong and Hualan, 2020). The authors hypothesized that SARS-CoV-2 could dissociate iron ions from porphyrin, resulting in a decreased affinity of Hb for oxygen (O_2_) and a decrease in O_2_ binding. They also speculated that this mechanism could lead to hemolytic anemia, and that some by-products could participate in the pathophysiology of the disease. Indeed, an excess in free heme has previously been shown to promote oxidative and inflammatory stress (Wagener et al., 2020).

Although they were not supported by any experimental validation, such as *in vitro* biochemical interaction, nor any clinical observation, these conclusions were largely relayed in the media and social networks. One response on the ChemRxiv platform, identifying presumed flaws in the computational analysis, did not get as much audience (Read, 2020). Several academics called for research investigating the interaction between Hb and SARS-CoV-2 (Chowdhury and Anwar, 2020; Wagener et al., 2020). The aim of this work was therefore to investigate whether the affinity of Hb for O_2_ was altered in COVID-19.

## Materials and Methods

### Patient Selection

This retrospective, observational, single-center study compared 100 patients with COVID-19 and 100 control patients. The COVID-19 group (group 1) included patients with positive SARS-CoV-2 polymerase chain reaction (PCR) and at least one blood gas analysis (BGA) collected in Avicenne University Hospital, Bobigny, France, between 2020/03/16 and 2020/04/12, either in emergency room (ER), general ward or intensive care unit (ICU). One hundred patients were randomly selected, with a 1:4 stratification on the number of collected BGAs (one BGA vs. ≥2 BGAs) in order to favor the inclusion of patients with ≥2 samples, so that the time-course of P_50_ could be evaluated.

The non-COVID-19 group was a historical “negative control” (group 2), it included patients with at least one BGA collected between 2019/03/01 and 2019/04/30. One hundred unmatched patients were randomly selected, with the same stratification. For each patient in COVID-19 and non-COVID-19 groups, 1–5 BGAs were selected (see below).

### Sample Selection

BGAs were made using an ABL90 FLEX or an ABL800 FLEX analyzer (Radiometer, Brønshøj, Denmark). Samples with fetal Hb (HbF) >20%, sickle Hb (HbS), or any technical problem (air bubbles, sample insufficiently shaken…) were discarded. To avoid a disproportion in the weight of each patient in the analysis, and to obtain samples collected at different levels of oxygen therapy, the number of samples was limited to 5 per patient in COVID-19 and non-COVID-19 groups, which were selected as follows: (a) first BGA in ER (if applicable); (b) first BGA in ward (if applicable); (c) first BGA in ICU (if applicable); (d) BGA after 8 ± 3 days of hospitalization or last BGA before death if the patient died before D_8_; (e) BGA after 15 ± 3 days of hospitalization or last BGA before death if the patient died before D_15_.

### Assessment of Hb Affinity

P_50_ is the oxygen partial pressure when Hb is 50% saturated with O_2_. It is negatively correlated with Hb affinity. For one BGA, a reliable value of P_50_ can be calculated when Hb saturation is <97% (BGAs with saturation ≥97% were not used for these analyses). To allow comparisons between samples, all P_50_ values were standardized for normal conditions (body temperature = 37°C; pH = 7.4; PCO_2_ = 40 mmHg). The normal value of P_50_ in these conditions is 26.8 mmHg (West and Luks, 2016). The oxyhemoglobin (HbO_2_) dissociation model was computed taking into account carboxyhemoglobin (HbCO) and methemoglobin (MetHb), using Hill’s model corrected by Dash in the (*p*,*s*) space of Roughton (Hill, 1921; Roughton and Darling, 1944; Dash et al., 2016): *s* is the combined O_2_ + carbon monoxide (CO) saturation (fHbO_2_ + fHbCO)/(1-fMetHb), and *p* = PO_2_ + M PCO where M is the Haldane ratio of affinities (Douglas et al., 1912). The curve was first displaced by all known effects (temperature, pH, PCO_2_), and the extra *p* scaling to match the BGA was measured. The same scaling was applied to the model P_50_ (computed for O_2_ saturation = 50%). This measured P_50_ was then scaled back to standard conditions using Dash’s model.

A raise in 2,3-diphosphoglycerate (2,3-DPG) can induce a decrease in Hb affinity for O_2_ (hence an increase in P_50_). 2,3-DPG concentration ([2,3-DPG]) was not routinely measured in our patients, however factors modulating [2,3-DPG] were assessed, such as Hb concentration ([Hb]), age, phosphatemia, and history of heart failure (de Verdier and Garby, 1972; Purcell and Brozović, 1974). As hydroxychloroquine is known to provoke methemoglobinemia (Hall et al., 1986), the relation between hydroxychloroquine and MetHb level in the COVID-19 group was also assessed.

### Model Validation

Two “positive control” groups for abnormal affinity were also used, to test if our model was able to detect clinically significant changes in Hb affinity in various conditions. All consecutive patients with HbCO ≥8%, starting from 2016/01/01, were included in the high HbCO group (group 3). One BGA per patient was selected. In this group, Hb was supposed to be normal, but the presence of an unusual amount of CO was expected to stabilize Hb in its relaxed R-state and to provoke an increase in Hb affinity for O_2_ (West and Luks, 2016).

Finally, the last group comprised 30 patients with homozygous HbSS sickle cell disease: SCD group (group 4). Data from all available BGAs with Hb saturation <97% were collected (from 2015/07/27 to 2020/12/08). The SCD group was used to assess if our model using Dash’s equations was still valid with abnormal Hb, as HbS affinity for O_2_ is decreased (Huynh-Moynot et al., 2011; Ribeil et al., 2017).

### Assessment of Hemolysis in Anemic Patients

Among COVID-19 patients, if at least one BGA showed a [Hb] ≤ 11 g.dl^–1^, blood smears and patient files were reviewed with a hemobiologist and the following data throughout the study period were gathered and analyzed: [Hb] on complete blood count (CBC), mean corpuscular volume (MCV), reticulocyte count, presence or absence of schistocytes, plasmatic concentrations of total and unconjugated bilirubin, lactate dehydrogenase (LDH), haptoglobin, ferritin, and C-reactive protein (CRP).

### Statistical Analysis

Demographic and blood gas characteristics were compared between COVID-19 and non-COVID-19 groups using χ^2^ test (qualitative variables), Mann-Whitney or unpaired *t*-test (quantitative variables, according to distribution). Differences between measured HbO_2_ and predicted HbO_2_ were assessed by unpaired *t* test. Before/after comparisons in the COVID-19 group (mechanical ventilation, [Hb]) were performed with paired *t-*test. Comparisons between ≥3 groups were assessed with Kruskal-Wallis test and Dunn’s multiple comparison test. Spearman correlation coefficient (*r*) was employed to examine the relation between P_50_ and [Hb], age or phosphatemia. For P_50_ time-course, two-way ANOVA was performed. A *p* < 0.05 was considered significant. Prism^®^ software was used (GraphPad Software Inc., San Diego, CA, United States).

## Results

### Study Population

All 100 COVID-19 patients being hospitalized or at least seen in ER, none of them was asymptomatic. Fever, dyspnea, cough and other classical COVID-19 symptoms were common. In the non-COVID-19 group, the most frequent diagnoses were infection, airway disease (chronic obstructive pulmonary disease, asthma, bronchiectasis…), interstitial lung disease or heart failure (Supplementary Table 1). Patients in both COVID-19 and non-COVID-19 groups did not differ statistically for age or sex ratio. COVID-19 patients were significantly heavier and more frequently non-smokers. They required higher O_2_ delivery at baseline (Table 1), and 80 finally necessitated O_2_ therapy at some point.

**TABLE 1:** Demographic and blood gas characteristics at baseline.

	**COVID-19 (*n* = 100)**	**Non-COVID-19 (*n* = 100)**	***p***
Age (years)	62 [48–72]	66.5 [52–76]	NS
**Sex**			
Male	70	69	
Female	30	31	
			NS
Body mass index* (kg.m^–2^)	29.5 [26.1–31.3]	25.4 [21.9–29.9]	**0.0002**
**Smoking history**			
Never smoker	51	39	
Former smoker	28	36	
Current smoker	4	16	
Not available	17	9	
			**0.009**
Pack-years^#^	20 [11–50]	30 [16–50]	NS
**Place of first sample**			
Emergency room	84	67	
Ward	12	22	
Intensive care unit	4	11	
			**0.017**
**Severity**			
Ambient air	51	70	
Low dose O_2_ (1-6 l.min^–1^)	35	21	
High dose O_2_ (≥7 l.min^–1^ or ventilation)	14	9	
			**0.023**
**Blood gas variables**			
Temperature (°C)	37.9 [37–38.7]	37 [36.5–37.1]	**<0.0001**
PO_2_ (mmHg)	75.8 [65–93]	72.6 [60.2–84]	*–*
PCO_2_ (mmHg)	35.7 [32–39.5]	38 [31.8–43.2]	*–*
pH	7.44 [7.41–7.47]	7.42 [7.38–7.46]	*–*
Hemoglobin (g.dl^–1^)	14 [12.6–15.2]	13.2 [11.4–14.7]	**0.006**
Oxyhemoglobin (%)	93.2 [90.4–95.5]	92.6 [88.6–94.4]	*–*
Oxygen content (ml.100 ml^–1^)	18.2 [16.4–20.1]	16.9 [14.1–19]	*–*
Carboxyhemoglobin (%)	0.9 [0.7–1.1]	1.3 [0.8–1.7]	*–*
Methemoglobin (%)	1.1 [1–1.2]	0.8 [0.6–1.1]	**<0.0001**
*P*_*50*_^⨎^ (mmHg)	26 [25.2–26.8]	25.9 [24–27.3]	NS

Data are presented as numbers, or medians and interquartile ranges between square brackets. Some blood gas variables were not statistically compared because of the patients receiving oxygen therapy. NS, non-significant.*BMI: the number of available values was 70 and 76 for each group, respectively.^#^Tobacco consumption: the number of available values was 28/32 and 44/52 for each group, respectively.^⨎^P_50_: the number of exploitable values (saturation < 97%) was 69 and 79 for each group, respectively. Bold values are significant *p*-values (i.e., under 0.05).

Fifty-five patients had displayed a HbCO ≥ 8% since 2016 and were included in the high HbCO group (median HbCO level: 9.4% [8.6–12.6]). The reason for HbCO elevation was tobacco consumption in 26 (47.3%), CO poisoning in 15 (27.3%), and undetermined in the 14 others (25.4%). Thirty patients were included in the SCD group. One hundred and twenty-one BGAs were analyzed in the present study, among which 106 were collected in a context of vaso-occlusive crisis (VOC) and/or acute chest syndrome (ACS). Other indications were: respiratory infection without ACS (*n* = 7), scheduled health check (*n* = 6), thoracic pain without VOC (*n* = 2). Demographic characteristics are presented in Supplementary Table 2.

### Blood Gas Characteristics

Among COVID-19 patients, 51 were on ambient air at baseline. Blood gases were analyzed from arterial sample for 48 of them and venous sample for the other 3. In the non-COVID-19 group, 70 patients were on ambient air at baseline, with 59 arterial and 11 venous samples (Table 2). Despite a trend for lower PO_2_ in the COVID-19 group, no statistical difference was seen for PO_2_, PCO_2_ or pH between COVID-19 and non-COVID-19 patients in ambient air. Median HbCO level was slightly, but significantly, lower in COVID-19 patients. On the contrary, median MetHb level was slightly, but significantly, higher in COVID-19 patients.

**TABLE 2:** Characteristics of the arterial blood gas analyses collected in ambient air at baseline.

	**COVID-19 (*n* = 48)**	**Non-COVID-19 (*n* = 59)**	***p***
Temperature (°C)	37.8 [36.9–38.3]	37 [36.5–37]	**<0.0001**
PO_2_ (mmHg)	71.5 [62.6–78.9]	76.1 [65.8–89.5]	NS
PCO_2_ (mmHg)	35.7 [32.1–38.4]	35.7 [30.2–38.7]	NS
pH	7.44 [7.42–7.46]	7.43 [7.41–7.48]	NS
Hemoglobin (g.dl^–1^)	14.5 [13.3–15.6]	13.4 [12.4–15]	**0.026**
Oxyhemoglobin (%)	91.9 [89.8–93.9]	93.3 [91–94.6]	NS
Oxygen content (ml.100 ml^–1^)	18.9 [16.9–20.6]	17.5 [15.9–19.2]	NS
Carboxyhemoglobin (%)	0.9 [0.7–1.2]	1.3 [0.8–2]	**0.002**
Methemoglobin (%)	1 [0.9–1.2]	0.7 [0.6–1]	**<0.0001**
P_50_* (mmHg)	26.1 [25.4–26.7]	26 [24.6–27.3]	NS

Data are presented as medians and interquartile ranges between square brackets. NS, non-significant.*P_50_: the number of exploitable values (saturation < 97%) was 41 and 56 for each group, respectively. Bold values are significant *p*-values (i.e., under 0.05).

### Hb Affinity in COVID-19 and Non-COVID-19 Groups

**FIGURE 1:**
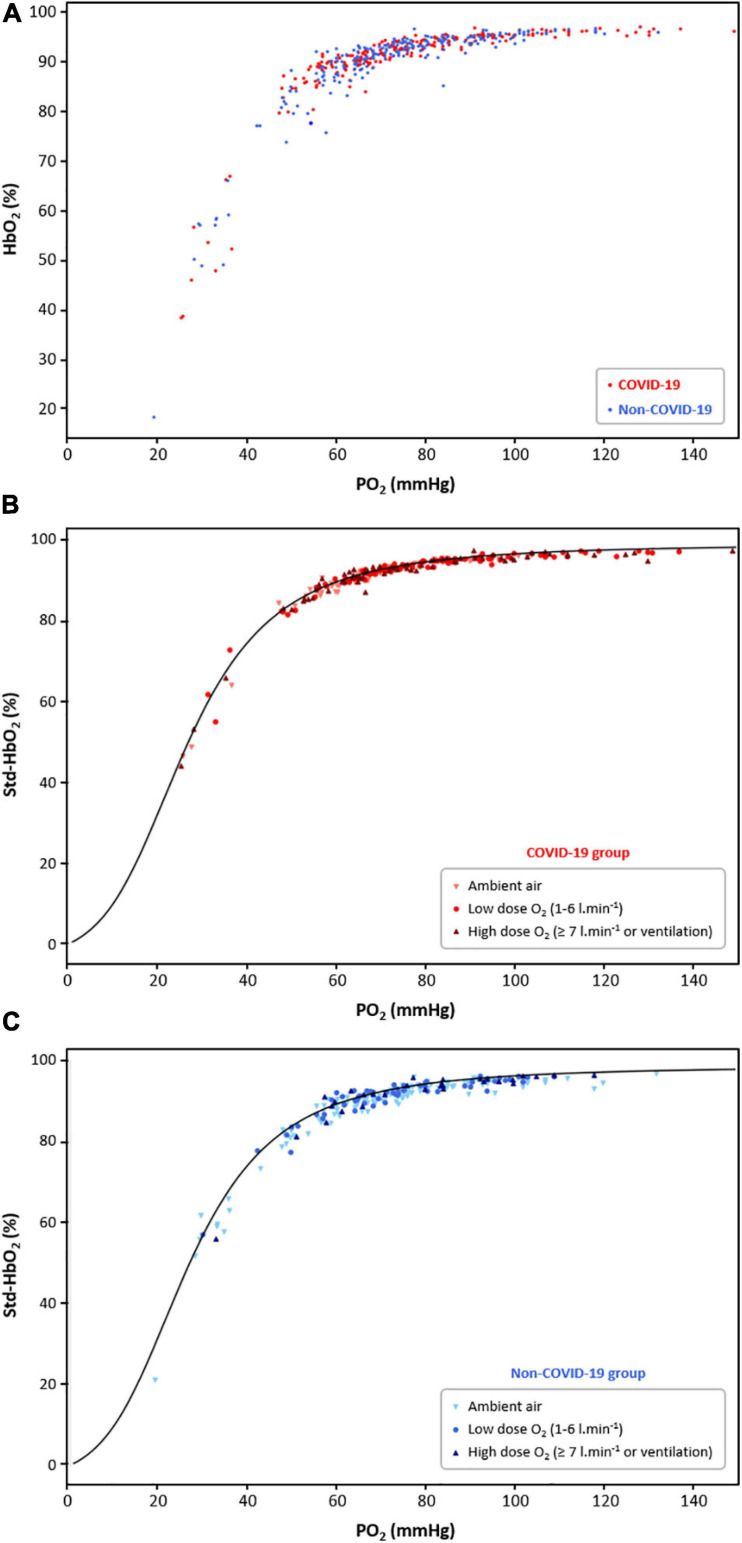
**(A)** Raw oxyhemoglobin (HbO_2_) in relation to PO_2_ in the COVID-19 group (red dots, 100 patients, 253 samples) and the non-COVID-19 group (blue dots, 100 patients, 221 samples). **(B)** Standardized HbO_2_ (Std-HbO_2_) in relation to PO_2_ in the COVID-19 group, according to the level of oxygen therapy (light red triangles: ambient air; medium red dots: O_2_ between 1 and 6 l.min^− 1^; dark red triangles: O_2_ ≥ 7 l.min^− 1^ or ventilation). Measured HbO_2_ was standardized for normal conditions (temperature = 37°C; pH = 7.4; PCO_2_ = 40 mmHg) in order to compare it to the predicted HbO_2_ given by the standard O_2_-Hb dissociation curve, represented in black. **(C)** Std-HbO_2_ for normal conditions in relation to PO_2_ in the non-COVID-19 group, according to the level of oxygen therapy (light blue triangles: ambient air; medium blue dots: O_2_ between 1 and 6 l.min^− 1^; dark blue triangles: O_2_ ≥ 7 l.min^− 1^ or ventilation).

A total number of 253 samples were selected for the 100 COVID-19 patients throughout the study period, and 221 in the non-COVID-19 group. Twenty-three COVID-19 patients and 27 non-COVID-19 patients had only 1 BGA. Raw HbO_2_ values (without standardization) in relation to PO_2_ in both groups are presented in Figure 1A, while HbO_2_ values standardized for normal conditions (Std-HbO_2_) in COVID-19 and non-COVID-19 groups are presented in Figures 1B,C, respectively. In both groups, mean difference between measured Std-HbO_2_ value and predicted HbO_2_ given by the standard O_2_-Hb dissociation curve was very low: −0.3 ± 0.7% (*p* = 0.73) and −1.1 ± 0.9% (*p* = 0.21), respectively. This low dispersion was observed at any given PO_2_ and whatever the level of oxygen therapy. Importantly, median P_50_ at baseline was not different between COVID-19 group (26 mmHg [25.2–26.8]) and non-COVID-19 group (25.9 mmHg [24–27.3]; *p* = 0.42) (Table 1). As expected, it was significantly lower in the high HbCO group (22.5 mmHg [21.6–23.8]) and significantly higher in the SCD group (29.3 mmHg [27–31.5] (*p* < 0.0001 for all comparisons). No correlation was found between P_50_ and age or phosphatemia (all correlation coefficients *r* < 0.15) or history of heart failure (*p* = 0.28) in both COVID-19 and non-COVID-19 groups. In the COVID-19 group, median MetHb level was significantly higher in the subgroup of samples collected in patients having received hydroxychloroquine (*n* = 74): 1.5% [1.2–1.8] vs. 1.1% [1–1.3] in the absence of hydroxychloroquine (*n* = 177) (*p* < 0.0001). Median P_50_ in these two subgroups was 25.5 mmHg [24.9–26.5] vs. 26.1 mmHg [24.6–27.3], respectively (*p* = 0.07).

**FIGURE 2:**
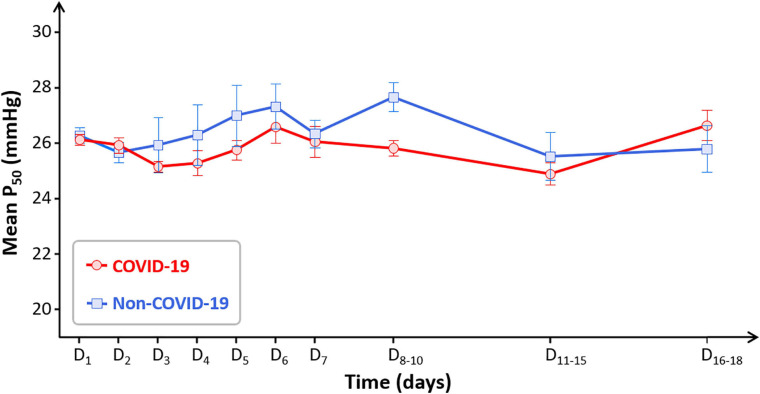
Time-course of mean P_50_ in the COVID-19 group (red circles) and the non-COVID-19 group (blue squares). Some days were regrouped to have sufficient number of samples (D_8__–__10_: *n* = 15 in the COVID-19 group and *n* = 10 in the non-COVID-19 group; D_11__–__15_: *n* = 11 in both groups; D_16__–__18_: *n* = 4 and *n* = 6, respectively). Data are presented as means and SE.

When considering P_50_ time-course between days 1 and 18, no significant difference was observed between COVID-19 and non-COVID-19 patients: no group effect nor time effect (*p* = 0.72) (Figure 2). Global P_50_ stability over time was similarly observed when considering only the most severe patients: eighteen COVID-19 patients necessitated mechanical ventilation, their median P_50_ was 25.7 mmHg [25.1–26] with mechanical ventilation and 26 mmHg [25.2–26.9] without (*p* = 0.19) (Supplementary Figure 1).

### Hb Affinity in the High HbCO Group

The graphical representations of raw HbO_2_ and Std-HbO_2_ in relation to PO_2_ show that Std-HbO_2_ reached an upper limit of 92.6% because of the competitive binding of CO to Hb (Supplementary Figures 2A,B). Contrary to COVID-19 and non-COVID-19 groups, mean difference between measured Std-HbO_2_ and predicted HbO_2_ was important in the high HbCO group: −8.6 ± 2.1% (*p* = 0.0001). Taking into account the combined saturation of Hb with O_2_ and CO (Std-SatO_2_ + CO), compared to the standard O_2_-Hb dissociation curve, a shift in the relation between combined saturation and PO_2_ was observed to the left, indicating a lower P_50_ and a greater Hb affinity for O_2_ (Supplementary Figure 2C). At last, when the partial pressure in CO (PCO) was also taken into account, the median difference between measured combined saturation and predicted combined saturation was reduced to −0.16 ± 1.65% (*p* = 0.92), indicating that our model was able to explain every kind of variation (Supplementary Figure 2D).

### Hb Affinity in the SCD Group

In the same line, mean difference between measured Std-HbO_2_ and predicted HbO_2_ was important in the SCD group: −5.3 ± 4.1% (*p* < 0.0001). As expected, a shift was observed to the right in the relation between Std-HbO_2_ and PO_2_, as compared to the standard O_2_-Hb dissociation curve (Supplementary Figure 3), indicating that our model was able to detect clinically significant changes in Hb affinity even in a group of patients with abnormal Hb (here, a rise in P_50_ with decreased Hb affinity). Taking into account combined Hb saturation (Std-SatO_2_ + CO) and PCO did not change those results. Of note, median P_50_ while taking hydroxycarbamide was significantly less elevated than without treatment: 28.2 mmHg [27–31.2] vs. 30 mmHg [26.9–31.9], respectively (*p* = 0.014). However, all samples were retained for analysis in the present study. On the other hand, the effect of transfusion on P_50_ could be assessed in a subgroup of 11 SCD patients: P_50_ decreased in only 7 of them after transfusion, and the mean difference in P_50_ before/after transfusion in the whole subgroup was −0.5 ± 1.7 mmHg (*p* = 0.38).

### Anemia and Hemolysis

Median [Hb] at baseline was significantly higher in the COVID-19 group than in the non-COVID-19 group (Table 1). Twenty-four COVID-19 patients displayed [Hb] ≤ 11 g.dl^–1^ at some point. Among them, 17 exhibited no biological sign of hemolysis, and the cause of anemia was undetermined for the other 7 (due to limited retrospective data). Among biological variables related to anemia, inflammation markers (ferritin, CRP) were the only significant differences between anemic patients from COVID-19 to non-COVID-19 groups (Supplementary Table 3).

Among the 24 anemic COVID-19 patients, before/after comparison of P_50_ between highest and lowest [Hb] was possible in 16 of them. At highest [Hb] (mean: 12.9 ± 1.4 g.dl^–1^), mean P_50_ value was 25.6 ± 0.9 mmHg; whereas it was 25.5 ± 1.2 mmHg at lowest [Hb] (mean: 9.3 ± 1.2 g.dl^–1^) (*p* = 0.6) (Supplementary Figure 4). Taking the whole COVID-19 population, no significant correlation was found between P_50_ and [Hb] (*r* = 0.19; *p* = 0.07). Similarly, no correlation between these two variables was found in the SCD group either (*r* = −0.06; *p* = 0.52).

## Discussion

Comparing P_50_ values and the distribution of HbO_2_/PO_2_ in relation to the standard O_2_-Hb dissociation curve in 100 COVID-19 patients and 100 non-COVID-19 patients, we found no argument to support the hypothesis that SARS-CoV-2 can be responsible for a clinically significant alteration of O_2_ binding to Hb, neither at baseline nor later in the disease course. In contrast, we were able to identify a shift in the relation between PO_2_ and HbO_2_, and variations in P_50_ value, in 55 patients with HbCO ≥8% (increased Hb affinity) and 30 patients with sickle cell disease (decreased Hb affinity, even more when they were not treated by hydroxycarbamide). Moreover, no COVID-19 patients displayed hemolysis stigma.

Due to the huge number of BGA samples collected in our institution during the study period (3,706 in March-April 2019 and 5,346 between 2020/03/16 and 2020/04/12, regardless of the diagnosis), a random draw of patients had to be performed. Despite our 1:4 stratification on the number of collected samples, the COVID-19 group finally comprised 23 patients with only 1 BGA, whereas they were 27 in the non-COVID-19 group. This was due to two facts: for some patients with multiple BGAs, only one sample finally met our selection criteria; conversely, for some patients that had only one BGA during the predefined period, we could analyze other samples collected before or after. Eventually, we were able to compare 253 BGAs from 100 COVID-19 patients with positive SARS-CoV-2 PCR, to 221 samples from 100 non-COVID-19 controls, providing extensive information about blood gases and Hb affinity for O_2_ in COVID-19.

Median P_50_ corrected for body temperature, pH and PCO_2_ at baseline was 26 mmHg [25.2–26.8] in the COVID-19 group vs. 25.9 mmHg [24–27.3] in the non-COVID-19 group. These values are slightly lower than the normal theoretical value of 26.8 mmHg, which is, however, calculated for normal HbCO and MetHb levels (West and Luks, 2016). In our study, non-COVID-19 patients displayed a slightly, but significantly, higher median HbCO level, which was consistent with the greater proportion of smokers in this group. On the contrary, COVID-19 patients displayed a slightly, but significantly, higher median MetHb level, probably secondary to the use of hydroxychloroquine in some of them, a drug which can potentially raise MetHb level (Hall et al., 1986). Both HbCO and MetHb are well known to increase Hb affinity for O_2_ and decrease P_50_ (Douglas et al., 1912; Darling and Roughton, 1942; Roughton and Darling, 1944). Moreover, [2,3-DPG] can be modified in diverse conditions: chronic hypoxia, alkalosis, heart failure and anemia can increase [2,3-DPG]; while acidosis, blood transfusion, polycythemia, hypophosphatemia and greater age can decrease it (de Verdier and Garby, 1972; Purcell and Brozović, 1974). Because [2,3-DPG] was not routinely measured in our institution, and although the main confounding factors were assessed in the present work (pH, history of heart failure, phosphatemia, age), it is possible that some of our patients displayed a decreased [2,3-DPG]. This could be another possible explanation for P_50_ values lower than 26.8 mmHg in our cohort. Anyway, the clinical significance of the effects of 2,3-DPG variation on oxygen affinity is thought to be minimal (Macdonald, 1977). Another limit of our study is that P_50_ was calculated in a blood gas analyzer, i.e., not measured. The technique to directly measure P_50_ is longer and not routinely performed: it consists in the exposure of a blood sample to an increasing partial pressure of oxygen and subsequent deoxygenation with nitrogen gas in a Hemox-Analyzer. However, as stated in the manufacturer reference manual, ABL FLEX analyzers can estimate a reliable value of P_50_ from a blood sample with saturation <97%. We proceeded as would do the blood gas analyzer, but we calculated standardized P_50_ using the equations validated by Dash (Dash et al., 2016). Indeed, our model was able to identify pathological P_50_ values in our “positive control” groups, even in the presence of abnormal Hb: most P_50_ values were lower than normal in the HbCO group, with a median P_50_ of 22.5 mmHg [21.6–23.8]; on the contrary most P_50_ values were higher than normal in the SCD group, with a median P_50_ of 30 mmHg [26.9–31.9] in untreated patients and 28.2 mmHg [27–31.2] in the ones receiving hydroxycarbamide. Moreover, P_50_ calculation by blood gas analyzers is, under certain conditions, routinely used by some referral centers in the diagnostic approach of hemoglobins with high O_2_ affinity (Orvain et al., 2017). Anyway, in the present study, the sample distribution of high HbCO and SCD groups was shifted from the standard O_2_-Hb dissociation curve, indicating a modified Hb affinity, whatever the potential lack of precision of P_50_ calculation in our model compared to the gold-standard. On the contrary, no clinically significant change of Hb affinity could be observed in COVID-19 patients, whose samples were clearly distributed on the standard dissociation curve, as for the non-COVID-19 control group.

Our findings are in line with the results of a British study conducted in only 14 critically ill COVID-19 patients, and 11 age- and sex-matched controls (Daniel et al., 2020). Mean P_50_, measured by Hemox-Analyzer, was not statistically different between both groups: 29 ± 2.3 vs. 28.5 ± 1.8 mmHg, respectively. The reasons why P_50_ values were higher than the normal theoretical value were not discussed. In another British study, mean P_50_ of 43 intubated and ventilated COVID-19 patients, retrospectively calculated from blood gas analyzer results, was 23.4 ± 3.13 mmHg, even significantly lower than a historical cohort of unmatched critically ill controls (24.6 ± 5.42 mmHg) (Vogel et al., 2020). The authors hypothesized that those low values could be explained by reduced [2,3-DPG], and that for some reason it was even more reduced in COVID-19 patients. Another possible cause was the use of samples with saturation ≥97% to calculate P_50_. At a cellular level, data are conflicting about the potential predisposition of impaired O_2_ transport during COVID-19 (Park et al., 2020; Thomas et al., 2020); but, to date, there is no biological evidence to support the hypothesis of Wenzhong and Hualan that SARS-CoV-2 could be responsible for a clinically significant alteration of Hb affinity for O_2_ (Wenzhong and Hualan, 2020). By the way, about 19% of COVID-19 patients are considered to display severe-to-critical pneumonia (Wu and McGoogan, 2020), with often profound hypoxemia which in no instance can be explained by altered Hb affinity (West and Luks, 2016).

Another claim in the preprint article of Wenzhong and Hualan (2020) was that SARS-CoV-2 could be responsible for hemolytic anemia. Indeed, potential causes of anemia are numerous and often intertwined in critically ill patients (hemodilution, iron deficiency by repeated blood sampling, surgical site bleeding or other invasive procedures, inflammation…) (Heming et al., 2011; Spinelli and Bartlett, 2016), particularly in such an inflammatory condition as COVID-19. In the present work, fever and dehydration could explain, at least in part, the higher median [Hb] in COVID-19 patients at baseline, compared to non-COVID-19 patients. Anyway, median [Hb] was normal at baseline, and although 24 COVID-19 patients later displayed anemia in the course of their disease, none of them exhibited obvious hemolysis. In a Chinese study comparing hematologic variables between critically ill COVID-19 and other COVID-19 patients not having required ICU, median [Hb] was normal at baseline in both groups, but the median [Hb] nadir was then lower in critically ill patients (11.1 g.dl^–1^ [10.2–11.9] vs. 13.6 g.dl^–1^ [12.7–15.1]) (Fan et al., 2020). In a literature review mostly analyzing data from Chinese centers, the authors stated that anemia was not a common laboratory finding in COVID-19 patients, but [Hb] tended to decline during hospitalization (Liu et al., 2020). At last, two meta-analyses showed that low [Hb] was associated with disease severity in COVID-19 (Alnor et al., 2020; Lippi and Mattiuzzi, 2020). Hemolysis was not mentioned in any of these articles. However, it cannot be excluded that occult intravascular hemolysis might occur at some level which could not be detected with classical biological signs, requiring more sensitive techniques such as detecting red blood cell microvesicles (Camus et al., 2015).

Several reports of acute hemolysis after SARS-CoV-2 infection were published, but not from direct viral action on Hb. Twelve patients presented with autoimmune hemolytic anemia (AIHA), among them 4 had B lymphoid malignancy, one had monoclonal gammapathy (Jensen et al., 2020; Lazarian et al., 2020; Lopez et al., 2020) and 2 had Evans syndrome (Li et al., 2020; Wahlster et al., 2020). Later, it was stated that AIHA could concern 12% of the subgroup of anemic COVID-19 patients (Algassim et al., 2021). Fourteen additional patients were described: five with paroxysmal nocturnal hemoglobinuria (Kulasekararaj et al., 2020; Pike et al., 2020) and 9 with previously undiagnosed glucose-6-phosphate dehydrogenase (G6PD) deficiency uncovered in context of acute hemolysis (Aguilar and Averbukh, 2020; Beauverd et al., 2020; De Franceschi et al., 2020; Kuipers et al., 2020; Maillart et al., 2020; Palmer et al., 2020; Sasi et al., 2020; Sgherza et al., 2020; Lopes et al., 2021). Indeed, infections are the most common trigger for hemolysis in G6PD-deficient individuals, and it is unclear if the use of chloroquine or hydroxychloroquine in these patients can worsen the phenomenon (Afra et al., 2020a, b).

In conclusion, the COVID-19 pandemic has greatly promoted preprint servers, with no less than 12,194 preliminary reports about COVID-19 hosted on arXiv platforms at the time of writing (MedRxiv, 2021). While it is a thrilling way to rapidly share information about the disease, the absence of conventional peer-review is at risk of spreading erroneous conclusions, sometimes amplified by the media and/or social networks (Smyth et al., 2020). The draft of Wenzhong and Hualan (2020) hypothesizing that SARS-CoV-2 could “attack” hemoglobin received quite a wide coverage and drew the public’s attention, as well as some academics’. However, the present study found no biological argument to think that Hb affinity for O_2_ is significantly altered during COVID-19, nor that COVID-19 can directly induce significant hemolytic anemia.

## Data Availability Statement

The raw data supporting the conclusions of this article will be made available by the authors, without undue reservation.

## Ethics Statement

The studies involving human participants were reviewed and approved by the Comité Local d’Ethique pour la Recherche Clinique Avicenne-Jean Verdier-René Muret (CLEA), Hôpitaux Universitaires de Paris-Seine-Saint-Denis (HUPSSD), Assistance Publique—Hôpitaux de Paris (AP-HP), Bobigny, France (#CLEA-2020-129). Written informed consent for participation was not required for this study in accordance with the national legislation and the institutional requirements.

## Author Contributions

TG, LS, and EA wrote the manuscript. TG, J-PR, and CP conceived and planned the study. EA processed the data (consistency checks, oxyhemoglobin dissociation model, P_50_ measurement, data standardization). EF extracted laboratory data. FC and KR reviewed the blood smears and files of anemic patients. TG performed the statistical analyses. All authors discussed the results and contributed to the final manuscript.

## Conflict of Interest

The authors declare that the research was conducted in the absence of any commercial or financial relationships that could be construed as a potential conflict of interest.

## Supplementary Material

Supplementary Figure 1 P_50_ with and without mechanical ventilation in the COVID-19 group. P_50_ values were standardized for normal conditions (temperature=37°C; pH=7.4; PCO_2_=40 mmHg). Before/after comparison was possible in 13 out of 18 COVID-19 patients having required mechanical ventilation, no significant difference was found (paired *t* test, *p*=0.38).

Supplementary Figure 2 **(A)** Raw oxyhemoglobin (HbO_2_) in relation to PO_2_ in the high HbCO group (55 patients, 55 samples). **(B)** Standardized oxyhemoglobin (Std-HbO_2_) in relation to PO_2_. Measured HbO_2_ was standardized for normal conditions (temperature=37°C; pH=7.4; PCO_2_=40 mmHg) in order to compare it to the predicted HbO_2_ given by the standard O_2_-Hb dissociation curve, represented in black. **(C)** Standardized combined saturation for oxygen and carbon monoxide (Std-Sat_*O*__2__+__*CO*_) in relation to PO_2_. **(D)** Std-Sat_*O*__2__+__*CO*_ in relation to combined partial pressure (PO_2_+M PCO).

Supplementary Figure 3 **(A)** Raw oxyhemoglobin (HbO_2_) in relation to PO_2_ in the SCD group (30 patients, 121 samples). **(B)** Standardized oxyhemoglobin (Std-HbO_2_) in relation to PO_2_. Measured HbO_2_ was standardized for normal conditions (temperature=37°C; pH=7.4; PCO_2_=40 mmHg) in order to compare it to the predicted HbO_2_ given by the standard O_2_-Hb dissociation curve, represented in black.

Supplementary Figure 4 Evolution of P_50_ in 16 anemic COVID-19 patients between highest and lowest hemoglobin concentration [Hb]. P_50_ values were standardized for normal conditions (temperature=37°C.; pH=7.4; PCO_2_=40 mmHg).

Supplementary Table 1 Main diagnosis in the non-COVID-19 group.

Supplementary Table 2 Demographic characteristics in high HbCO and SCD groups.

Supplementary Table 3 Biological data related to anemia in patients from all groups with hemoglobin concentration ≤ 11 g.dl^–1^.
