# Supplementary figures and images for: The Affinity of Hemoglobin for Oxygen Is Not Altered During COVID-19
Source: Front Physiol. 2021 Apr 12;12:578708. doi: 10.3389/fphys.2021.578708 (PMC8072381; doi:10.3389/fphys.2021.578708)

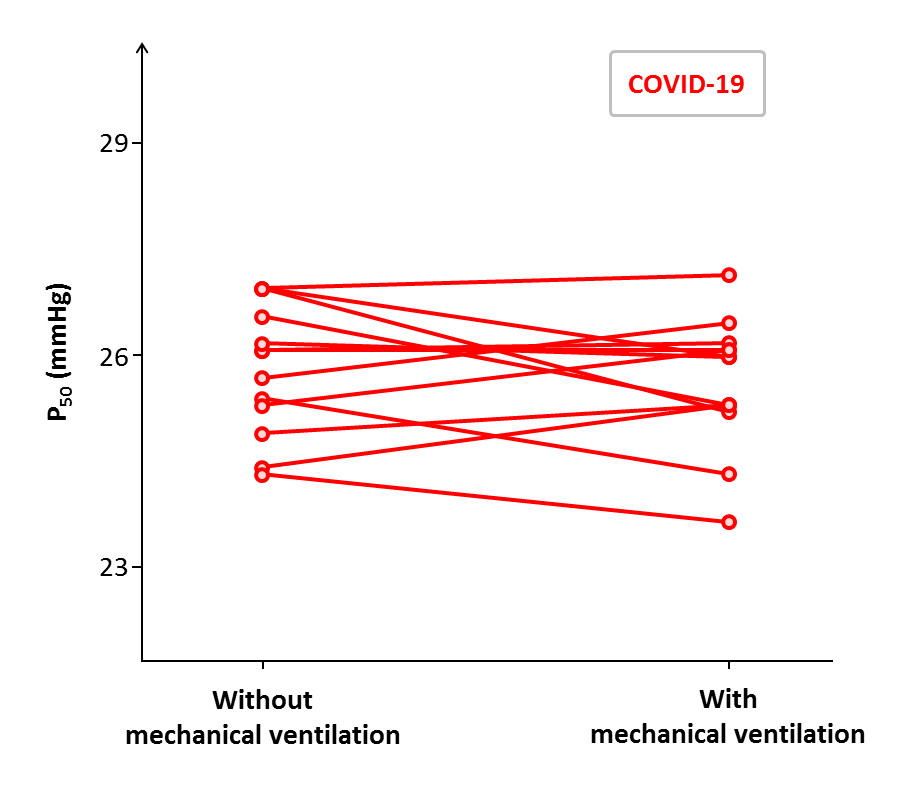

Supplement: Supplementary Figure 1 — P50 with and without mechanical ventilation in the COVID-19 group. P50 values were standardized for normal conditions (temperature=37°C; pH=7.4; PCO2=40 mmHg). Before/after comparison was possible in 13 out of 18 COVID-19 patients having required mechanical ventilation, no significant difference was found (paired t test, p=0.38). [file Image_1.TIF]

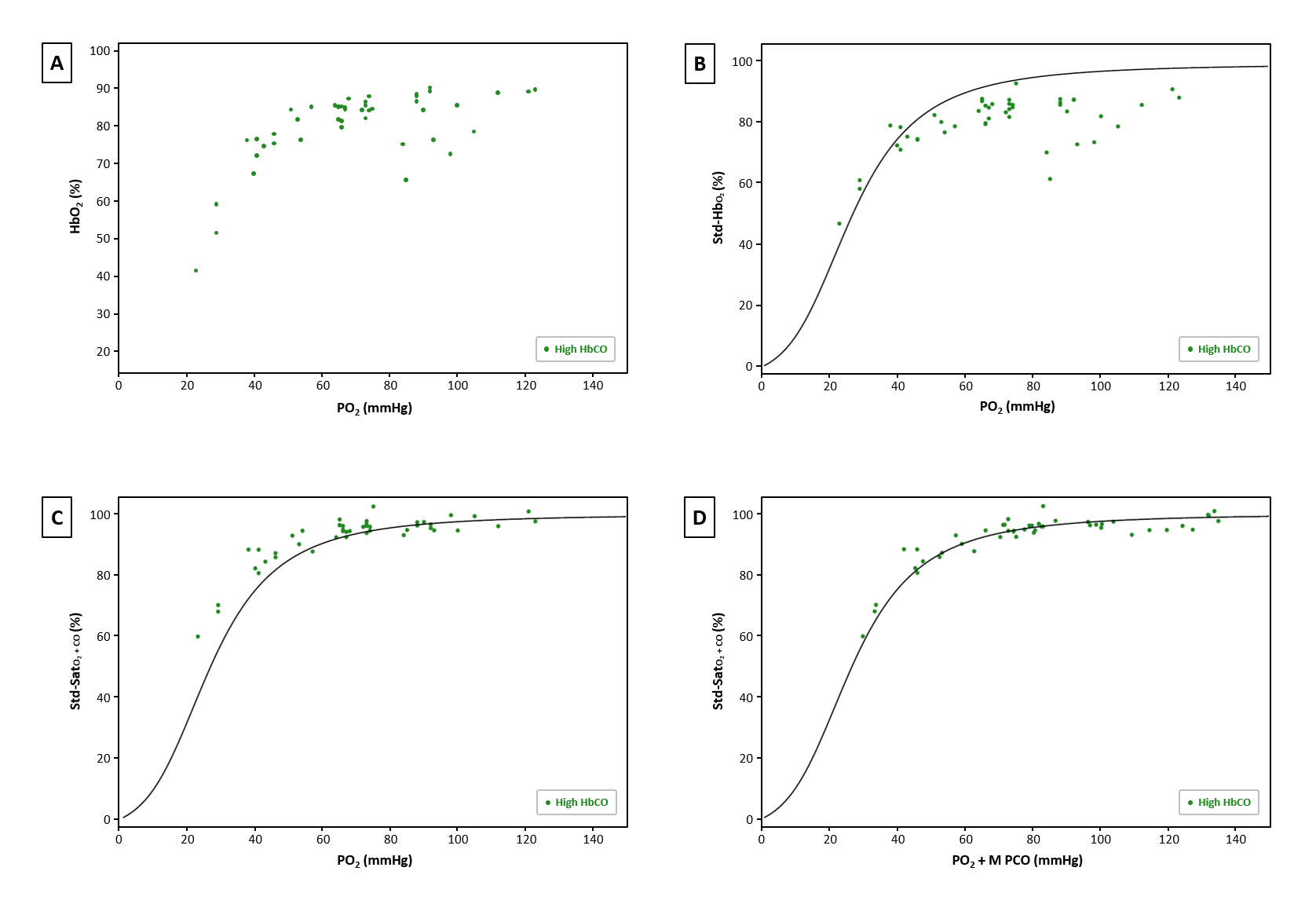

Supplement: Supplementary Figure 2 — (A) Raw oxyhemoglobin (HbO2) in relation to PO2 in the high HbCO group (55 patients, 55 samples). (B) Standardized oxyhemoglobin (Std-HbO2) in relation to PO2. Measured HbO2 was standardized for normal conditions (temperature=37°C; pH=7.4; PCO2=40 mmHg) in order to compare it to the predicted HbO2 given by the standard O2-Hb dissociation curve, represented in black. (C) Standardized combined saturation for oxygen and carbon monoxide (Std-SatO2+CO) in relation to PO2. (D) Std-SatO2+CO in relation to combined partial pressure (PO2+M PCO). [file Image_2.TIF]

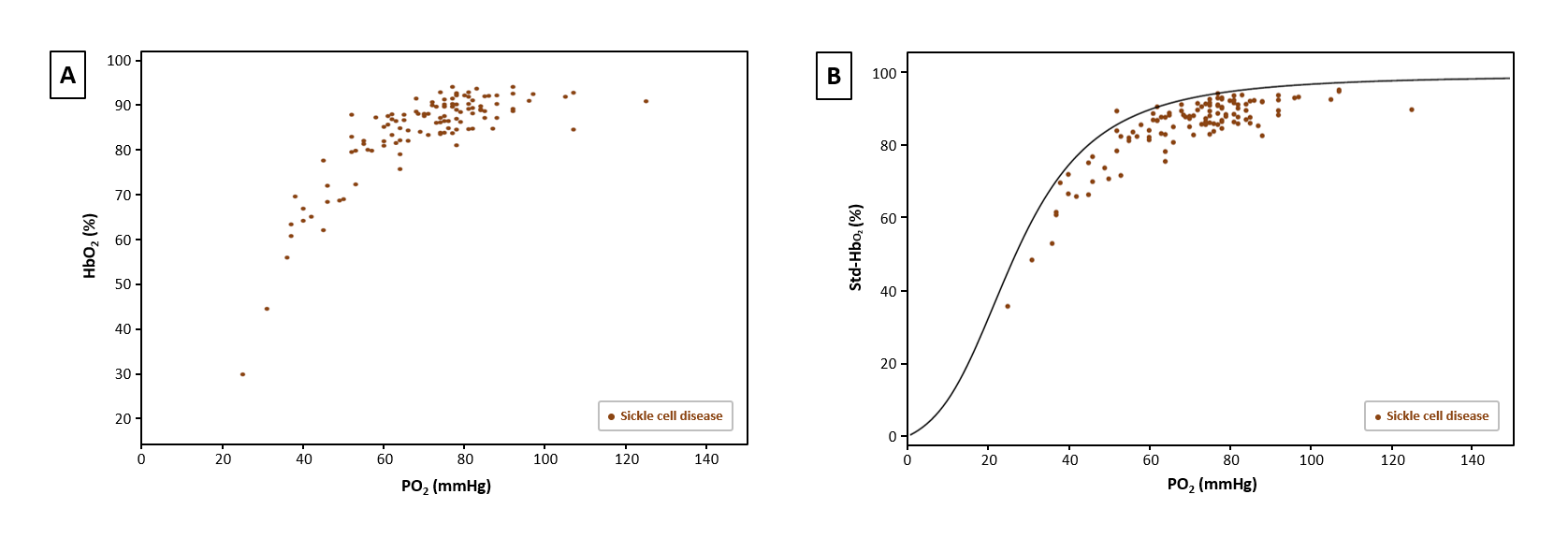

Supplement: Supplementary Figure 3 — (A) Raw oxyhemoglobin (HbO2) in relation to PO2 in the SCD group (30 patients, 121 samples). (B) Standardized oxyhemoglobin (Std-HbO2) in relation to PO2. Measured HbO2 was standardized for normal conditions (temperature=37°C; pH=7.4; PCO2=40 mmHg) in order to compare it to the predicted HbO2 given by the standard O2-Hb dissociation curve, represented in black. [file Image_3.TIF]

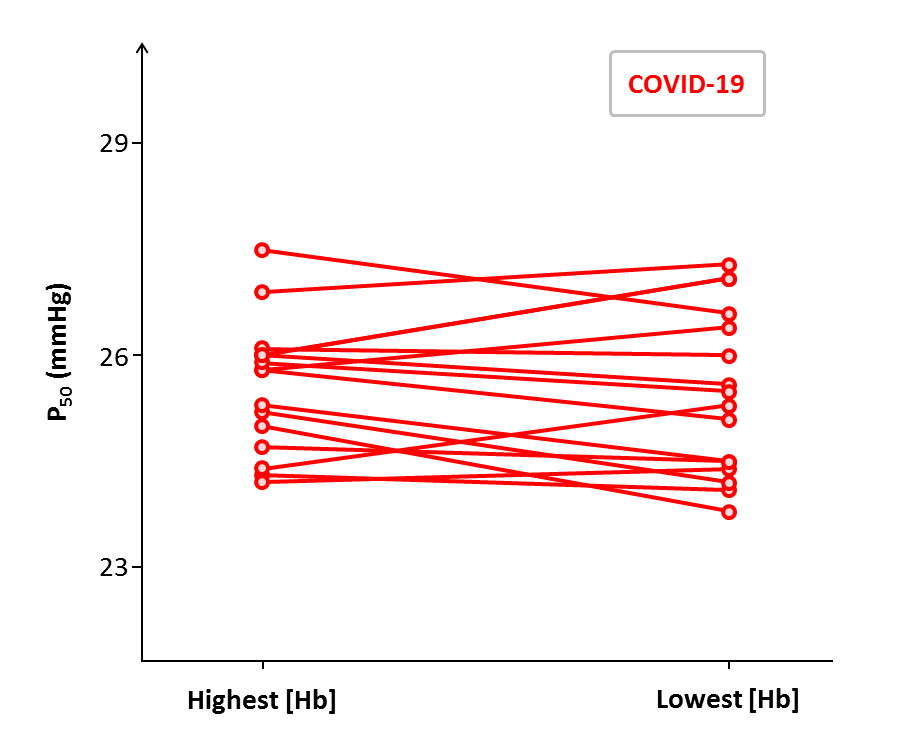

Supplement: Supplementary Figure 4 — Evolution of P50 in 16 anemic COVID-19 patients between highest and lowest hemoglobin concentration [Hb]. P50 values were standardized for normal conditions (temperature=37°C.; pH=7.4; PCO2=40 mmHg). [file Image_4.TIF]
