# Supplementary material for: The Affinity of Hemoglobin for Oxygen Is Not Altered During COVID-19
Source: Front Physiol. 2021 Apr 12;12:578708. doi: 10.3389/fphys.2021.578708 (PMC8072381; doi:10.3389/fphys.2021.578708)
Supplement: Supplementary Table 1 — Main diagnosis in the non-COVID-19 group. [file Table_1.DOCX]

| **Supplementary Table S1 \|** Main diagnosis in the non-COVID-19 group. | |
| --- | --- |
| Infection  Airway disease  Interstitial lung disease  Cardiac failure  Perioperative period  Pleural disease  Malaise  Neoplasia  Thrombo-embolic disease  Thoracic pain  Renal failure  Medication overdose  Other | 18  14  14  12  7  6  5  4  4  3  3  3  6 |
