# Supplementary material for: The Affinity of Hemoglobin for Oxygen Is Not Altered During COVID-19
Source: Front Physiol. 2021 Apr 12;12:578708. doi: 10.3389/fphys.2021.578708 (PMC8072381; doi:10.3389/fphys.2021.578708)
Supplement: Supplementary Table 2 — Demographic characteristics in high HbCO and SCD groups. [file Table_2.DOCX]

| **Supplementary Table S2 \|** Demographic characteristics in high HbCO and SCD groups. | | |
| --- | --- | --- |
|  | **High HbCO**  **(n = 55)** | **Sickle cell disease**  **(n = 30)** |
| **Age** (years) | 46 [34−57] | 27 [22−30] |
| **Sex**  **Male**  **Female** | 34 (61.8%)  21 (38.2%) | 17 (56.7%)  13 (43.3%) |
| **Body mass index*** (kg.m^-2^) | 24.9 [21.9−28.1] | 23.1 [19.3–25.2] |
| **Smoking history**  **Never smoker**  **Former smoker**  **Current smoker**  **Not available**  **Pack-years**^#^ | 2 (3.6%)  2 (3.6%)  34 (61.8%)  17 (31%)  40 [23−70] | 17 (56.7%)  0  9 (30%)  4 (13.3%)  2 [2−5] |

Data are presented as numbers and proportions between parentheses, or medians and interquartile ranges between square brackets. HbCO: carboxyhemoglobin; SCD: sickle cell disease.

* BMI: the number of available values was 25 and 29 for each group, respectively.

^#^ Tobacco consumption: the number of available values was 29/36 and 9/9 for each group, respectively.
