# Supplementary material for: The Affinity of Hemoglobin for Oxygen Is Not Altered During COVID-19
Source: Front Physiol. 2021 Apr 12;12:578708. doi: 10.3389/fphys.2021.578708 (PMC8072381; doi:10.3389/fphys.2021.578708)
Supplement: Supplementary Table 3 — Biological data related to anemia in patients from all groups with hemoglobin concentration ≤ 11 g.dl–1. [file Table_3.DOCX]

| **Supplementary Table S3 \|** Biological data related to anemia in patients from all groups with hemoglobin concentration ≤ 11 g.dl^-1^. | | | | | |
| --- | --- | --- | --- | --- | --- |
|  | **COVID-19**  **(n = 24)** | **Non-COVID-19**  **(n = 36)** | **Sickle cell disease**  **(n = 30)** | ***p*** |  |
| **Hemoglobin** (g.dl^-1^)  **Mean corpuscular volume** (fl)  **Reticulocytes** (10^9^.l^-1^)  **Total bilirubin** (µmol.l^-1^)  **Unconjugated bilirubin** (µmol.l^-1^)  **Lactate dehydrogenase** (U.l^-1^)  **Haptoglobin** (g.l^-1^)  **Ferritin** (µg.l^-1^)  **C-reactive protein** (mg.l^-1^) | 9.3 [8−10.4]  85 [79.5−94]  61 [34−101]  12 [8−19]  3 [2−10]  401 [352−655]  2.1 [1.36−3.93]  1472 [500−2618]  239 [136−346] | 9.1 [8.3−10]  85.3 [82.1−90.6]  89 [64−128]  9 [6−20]  6 [4−12]  576 [453−711]  2.58 [1.45−4.29]  220 [101−844]  151 [31−253] | 8.9 [8−9.4]  91.6 [77.6−94.1]  428 [358−563]  65 [37−107]  43 [25−78]  1143 [890−1366]  < 0.1 [< 0.1 − < 0.1]  200 [110−454]  52 [12−149] | *NS*  *NS*  ***< 0.0001***  ***< 0.0001***  ***< 0.0001***  ***< 0.0001***  ***< 0.0001***  ***< 0.0001***  ***< 0.0001*** |  |

The number of anemic patients was 24/100 in the COVID-19 group, 36/100 in the non-COVID-19 group and 30/30 in the sickle cell disease (SCD) group.

Only 1/55 patient was anemic in the high carboxyhemoglobin (HbCO) group, with the following data: hemoglobin concentration = 7.5 g.dl^-1^, mean corpuscular volume = 84.8 fl, reticulocyte count = 68.10^9^.l^-1^, total bilirubin concentration = 23 µmol.l^-1^, unconjugated bilirubin concentration = 12 µmol.l^-1^, lactate dehydrogenase concentration = 649 U.l^-1^, haptoglobin concentration = 1.03 g.l^-1^, ferritin concentration = 1237 µg.l^-1^, C-reactive protein concentration = 54 mg.l^-1^.
